# Variation in Trauma Team Response Fees in United States Trauma Centers: An Additional Undisclosed Variable Cost in Trauma Care

**DOI:** 10.7759/cureus.21776

**Published:** 2022-01-31

**Authors:** Michael M Neeki, Jan Serrano, Fanglong Dong, Mason H Chan, Danny Fernandez, Arianna S Neeki, Richard Vara, David T Wong, Rodney Borger, Louis Tran

**Affiliations:** 1 Emergency Medicine, Arrowhead Regional Medical Center, Colton, USA; 2 Trauma, Arrowhead Regional Medical Center, Colton, USA; 3 Emergency Medicine, California University of Science and Medicine, Colton, USA; 4 Surgery, Arrowhead Regional Medical Center, Colton, USA

**Keywords:** medical bankruptcy, activation fees, trauma centers, trauma care, trauma cost

## Abstract

Background: The rising costs associated with trauma care in the United States is an important topic in today’s healthcare environment. Factors such as innovations in technology, increasing governmental and organizational regulations, and the specialization of care have led to increasing costs to the patient. A component of trauma cost is the one-time trauma team response fee (TTRF). The determination process of the TTRF’s dollar amount is elusive as no apparent standardized process exists and the literature is scant regarding this aspect of trauma care.

Methods: A nationwide cross-sectional convenience sample was conducted using SurveyMonkey. Surveys were sent to 525 trauma centers in the continental United States, including Alaska and Hawaii, between October 8, 2019 and March 11, 2020. Additionally, hospital medical directors and trauma medical directors were queried on their knowledge of their facility’s TTRF amount.

Results: Only 46 out of 525 trauma centers, or 8.8% of those surveyed shared their scheduled fees. Comparisons of TTRFs among different trauma centers, activation levels, and geographical locations were not statistically significant.

Conclusions: Understanding the true costs of trauma care and fees for patients in the United States remains elusive due to inadequate data and low response rates. Trauma centers struggle to maintain financial viability as regulatory agencies and the public push for transparency of TTRFs. Collaboration between trauma centers and regulatory agencies is needed to ensure a balance between providing quality trauma care with justified associated charges and financial sustainability.

## Introduction

Trauma is one of the leading causes of death and trauma care is consistently ranked as one of the largest contributors to total healthcare costs in the United States [1,2]. The formalization of trauma centers in the 1980s added to the cost with 24-hour staffing of various medical specialists, equipment, and affiliated educational programs [3,4]. Although many hospitals accepted the added costs when choosing to become a trauma center, institutions could only use standard emergency department billing codes while rendering care for more complex trauma patients. As a result, many trauma centers terminated their trauma programs because they could not sustain the rising costs of providing specialized trauma care [3].

In 2002, the National Uniform Billing Committee approved the trauma activation fee (TAF) code (UB-04 revenue code 68X), which provided the much needed financial support for trauma centers through patient reimbursement [5-8]. Of note, this additional billing code can only be used for trauma patients arriving by ambulance [6]. The TAF billing code entails the initial and one-time trauma team response fee (TTRF) and is based on tiered trauma team response criteria [9,10]. Since not all trauma patients arrive by ambulance, it has been suggested that the discrepancy in billing practices may have resulted in the compulsive billing to offset costs of non-ambulance trauma patients [7,11]. To our knowledge, only a few local governments provided financial support, such as Los Angeles county parcel tax, to offset the costs of trauma centers.

The overall TTRF determination may consist of many variables ranging from local and national trauma designation to human and infrastructure costs [7,12]. Sparse national data are available on the process of how TTRFs are determined. Moreover, no standardized process on justified billing practices appears to be in place [13]. The following two cases provide an example of the disparity of TTRFs within one trauma center. The first case was an 18-month-old child who fell 3 feet off a bed with minimal injuries and was observed for about 3 hours without any radiographic studies. The second case was an adult patient who fell off a rock wall in a gym, requiring ankle surgery. The TTRF was $15,666 for both cases [13]. Both cases made the news and the backlash was swift as the public was enlightened on the astronomical costs of trauma care.

Increasing healthcare costs along with high numbers of uninsured and underinsured resulted in medical care costs as the number one cause of bankruptcy in the United States [14,15]. Public awareness of exorbitant trauma care costs has further supported demands for transparency in trauma billing practices. Legislative efforts in Connecticut and Florida have begun requiring disclosure of TTRFs. As part of consumer protection and reduction of healthcare expenditures, trauma centers in Connecticut are required to disclose TTRF [16,17]. As disclosures in medical billing practices become more common, additional regulations from state and federal agencies for TTRFs may be anticipated. This study aimed to investigate the variation of TTRF among all levels of trauma centers (Level I-IV) in different geographic regions in the United States. Regions were defined as Midwest, West, South, and Northeast based on the US Census Bureau region definitions [18].

A preprint of this article was previously posted to the Research Square server on August 19, 2021 (https://www.researchsquare.com/article/rs-777486/v1).

## Materials and methods

This was a cross-sectional convenience sample survey study utilizing the online survey software SurveyMonkey. The list of trauma centers was obtained from the Society of Trauma Nurses. Surveys were sent to 525 trauma centers in the continental United States, including Alaska and Hawaii. The SurveyMonkey link was emailed to the following positions: hospital medical director, trauma program director, trauma program manager, trauma nurse coordinator, director of emergency services, pediatric trauma coordinator, and trauma operations manager. Responses obtained from October 8, 2019 through March 11, 2020 were included in this study. Trauma centers not responding to the questionnaire were ultimately excluded from the final analysis.

Trauma centers included in the study were classified by region, trauma designation level, presence of academic programs, and verification status by American College of Surgeons (ACS). Questions were created by the investigators to capture the costs of a full or partial trauma team response, patient volume, and knowledge of the dollar amount of TTRFs by the trauma and hospital medical directors (Table 1). For this study, we classified tier A as the highest acuity response and tier C as the lowest acuity response (Table 1) [17].

**Table 1:** Survey Questions ACS= American College of Surgeons

1.	Hospital Name (Optional)
2.	City, State
3.	Trauma Level?
4.	Resident Training Program?
5.	ACS Verified?
6.	Base Cost for Trauma Team Activations Highest Tier Level (Most Critical) Second Tier Level Third Tier Level (Trauma Consultation)
7.	Number of Patients in Trauma Registry for 2018?
8.	Does Your Trauma Medical Director Know The Base Cost?
9.	Does Your Hospital Medical Director Know The Base Cost?

This study was approved by the Institutional Review Board at Arrowhead Regional Medical Center (ARMC). ARMC is a 456-bed acute care teaching facility and ACS-verified level II trauma center located in San Bernardino County, California, USA. The ARMC emergency department is one of the busiest in the state of California with more than 100,000 visits and over 3000 adult traumas annually [19].

All statistical analyses were conducted using the statistical software SAS for Windows version 9.3. Descriptive statistics were presented as frequencies and proportions for categorical variables, along with medians and interquartile range for continuous variables. Kruskal-Wallis tests were conducted to assess if there was a statistical difference among various trauma levels. All statistical analyses were two-sided. p-Value ≤0.05 was considered to be statistically significant.

## Results

A total of 46 responses were received and included in the final analysis with a response rate of 8.8%. More than half (n=26, 56.5%) had academic training programs, and 71.7% (n=33) were ACS-verified trauma centers. The highest number of responses came from the South region (37%, n=17). Of the respondents, 56.5% (n=26) of trauma medical directors and 41.3% (n=19) of hospital medical directors were aware of the cost of the TTRF (see Table 2).

**Table 2:** Survey Participants' Demographic Summary ACS=American College of Surgeons

	N	Percent
Trauma Level		
1	22	47.8
2	12	26.1
3	12	26.1
Resident Training Program		
No	20	43.5
Yes	26	56.5
ACS Verified		
No	13	28.3
Yes	33	71.7
Region		
Midwest	9	19.6
Northeast	9	19.6
South	17	37
West	11	23.9
Does your Trauma Medical Director Know the Base Trauma Cost?		
Yes	26	56.5
No	19	41.3
Unknown	1	2.2
Does Your Hospital Medical Director Know the Base Trauma Cost?		
Yes	19	41.3
No	25	54.4
Unknown	2	4.4

The TTRFs ranged from $200 to $23,000, with the highest fee reported in the West region. The widest range within the same tier and trauma center level was $9,840.90, among Level II trauma centers with a Tier B trauma response, also reported in the West region. Table 3 presented the analysis results. Overall, there is no statistically significant difference among the four regions on the highest-level response cost (all p-values >0.05), and second tier level response cost (all p-values >0.05).

**Table 3:** Reported Trauma Team Response Fees by Region* *All values were reported as median and corresponding first and third quartiles inside the parenthesis. All units were in US dollars. **Only one or two values were reported in the data. The data came from the actual numbers reported by the respondents. Median and corresponding first and third quartiles were not available for computation. These values were not used for the comparison of median cost among the geographic locations.

	Midwest	Northeast	South	West	p-Value
Tier A – high acuity response					
Level I Trauma Center	8805 (5585, 9177)	14,051**	8942 (6000, 10,600)	13879, 23,000**	0.3271
Level II Trauma Center	4150**	No data	5569, 13,000**	12,000 (8159.1, 18,000)	N/A
Level III Trauma Center	3300 (1143.8, 4356)	5000 (600, 6800)	4700 (3250, 6200)	1500**	0.7408
Tier B – Intermediate acuity response					
Level I Trauma Center	6368 (3767, 7745)	13,519**	7946 (5000, 9990)	8311, 13,000**	1
Level II Trauma Center	2459**	No data	3898, 8000**	5152 (5000, 6416)	N/A
Level III Trauma Center	1143, 4229**	3100**	3500 (1900, 4800)	1500**	N/A
Tier C – low acuity response					
Level I Trauma Center	2608, 3600**	8851**	3200, 3930**	6000**	N/A
Level II Trauma Center	200**	No data	2784**	3209, 6600**	N/A
Level III Trauma Center	No data	No data	337**	No data	N/A

## Discussion

Previous analysis of TTRF found little rationale in billing practices [13]. Hospitals are expected to provide qualified trauma care while costs are rising and revenue sources are limited. The ACS recommendations and policies have emphasized overtriage as it has minimal adverse medical consequences, while undertriage may result in preventable mortality or morbidity. However, the ACS acknowledges that this may result in excessive costs and burden for trauma centers [20]. Adding to the financial burden for trauma centers is the low collection rate of trauma charges. Fortune and colleagues reported that on average only 28.8% of trauma charges are collected [7]. Furthermore, while comparing two different trauma centers, the authors noted that revenue can be enhanced with compulsive billing and an emphasis on pre-admission designation of trauma patients [7].

It has also been suggested that hospitals may charge more to privately insured patients to offset the loss from caring for uninsured or underinsured patients [13,21]. In addition, there have been growing concerns that trauma centers are using alternative billing practices to increase revenue at the patient’s expense [13]. Patients are, therefore, left to pay catastrophic medical bills or copayments not covered by their insurance companies.

To address the national crisis in substandard trauma care, trauma centers were developed and expanded in the 1980s. Currently, there are 2352 designated trauma centers in the continental United States, including Alaska and Hawaii. It has been suggested that the increase in the number of trauma centers and specialized trauma care programs may directly contribute to the overall cost of healthcare in the United States. Even among the insured patients, the rising healthcare cost has been burdensome, and medical bankruptcy is now the leading cause of bankruptcy in the United States [14,15].

The true cost of trauma care is difficult to calculate. While each trauma patient requires individualized care, a team of trauma care specialists and subspecialists must be maintained on a call panel in each trauma center around the clock. Additionally, trauma centers also need to provide the infrastructure to ensure that appropriate care can be delivered to all trauma patients who present to the hospital. Ashley et al. noted that in 2019 the annual trauma team readiness cost was estimated to be over $10 million for each Level I trauma center, and nearly $5 million for each Level II trauma center in the state of Georgia [11]. This readiness cost is expended regardless of patient volume or insurance status [11].

All trauma centers are required to pay an initial and annual designation fees, which often vary among different counties and states [4,22]. Trauma centers that are verified by the ACS also incur an additional cost of annual assessment by the ACS. Other hidden costs may also include the maintenance of formal academic training programs. The Association of American Medical Colleges estimated that the direct training cost for teaching hospitals is approximately $19.2 billion annually, of which only $3.98 billion is supported by Medicare payment with no increases since 1997 [23].

Without adequate support from local, state, or national governments, the financial burden falls directly on the trauma centers. In response, the TTRF was developed to allow hospitals to recuperate some of the trauma-related costs whenever their trauma team is called to action. The TTRF is tiered and is correspondingly based on the tiered response criteria developed by the ACS. The cost of maintaining the minimal standards is, therefore, often passed to the patients. This has created a perception of inconsistent and discrepant trauma billing practices. As a result, public outcry had prompted legislative actions demanding for transparency in trauma billing practices, a concern echoed also by the ACS [24].

Historically, transparency in the disclosure of trauma cost has not been common, and proper data remain elusive. A national survey on trauma response fees among members of the National Foundation for Trauma Care/Trauma Center Association of America in 2007 had a response rate of 26.3% [5]. They reported TAFs ranging from $837 to $24,964. Interestingly, they also noted that, on average, Level II trauma centers were charging more than Level I trauma centers [5]. While a wide range is expected when comparing TTRF from trauma centers in different regions of the country, this difference has also been noted within each individual state. Mabry and colleagues examined the financial impact in the Arkansas statewide trauma system, and reported response fees ranging from $450 to $1689 [25]. A similar study in Florida noted a range from $197 to $66,000 [26].

While the response rate for our study is low at 8.8%, some observations can still be made. A wide range among the national TTRF still continues to exist. Among our respondents, the TTRF ranged from $200 to $23,000. Even among the same tiered response (Tier A) within the same trauma center level (Level II, West region), there was a difference of nearly $10,000. Unfortunately, the poor response rate prevented us from reaching any statistical significance.

Attempts to balance responsible financial billing and maintaining viability is an ongoing concern for trauma centers. This is more complicated as demands for transparency in trauma billing are on the rise. Although TTRFs continue to increase steadily, it is unclear how the amount is derived [25]. In our study, only 41.3% of hospital medical directors and 63% of trauma medical directors were aware of the fee amount of the TTRFs. The low percentage of medical directors who were aware of the TTRF in conjunction with the low response rate to our study demonstrated a concern for the lack of transparency in trauma billing, either intentional or not. In addition, a prior study reported that very few emergency physicians (EPs) can demonstrate appropriate cost awareness of routine medical care in the emergency department. Gandhi et al. noted that only 14% of EPs were able to correctly estimate the healthcare cost incurred by the patients [27]. Physicians should be educated to gain a better understanding of the billing practices to better serve their patients. Having transparency will provide trauma leadership with crucial information to monitor for possible over-triaging and over-billing. While eliminating all overtriage is not medically feasible, continuous oversight from the trauma medical director may aid in reducing unnecessary overbilling.

Trauma centers are critical to regionalized trauma networks (RTNs) and their sustained financial viability ensures high-quality care and safety for their communities [28]. These RTNs encompass the collaborative effort of trauma centers along with their partner agencies to provide a cost-effective coordinated comprehensive plan that optimizes high-quality care for injured patients [28]. In order to contain the rising cost of the TTRF, adequate financial support from federal and state governments is needed. To help justify the cost of trauma billing, educating the public is also important. Once the public understands the actual cost of maintaining a viable trauma center, it may rally their support for improved funding from responsible government agencies.

Limitations of this study include a low response rate from trauma centers and incomplete data, specifically on charges. Additionally, the survey questions did not include information on who determines the TTRF and the specific factors, such as cost of living adjustment, that influence the regional billing practices. Future studies should include identification of specific processes and factors that influence how TTRFs are determined.

## Conclusions

Trauma centers struggle to maintain financial viability as healthcare costs increase and limited options are available to offset those costs. Regulatory and public awareness of these increasing TTRF has resulted in a push for transparency. Improving hospital and trauma leadership knowledge and participation in setting the TTRF along with federal and state financial supports are needed to aid in the prevention of disparity in patient charges and assuring quality of trauma care for the public. In addition, vigilant efforts are needed in patient advocacy to ensure that all patients receive quality trauma care with justified associated charges.
